# Empowering partnership: key lessons from the co-development of patient-oriented research with parents, researchers, and healthcare professionals

**DOI:** 10.3389/frhs.2025.1624820

**Published:** 2025-08-08

**Authors:** Jacqueline M. Wilson, Seija K. Kromm, Christine Johns, Michelle L. Neraasen, Tapuwa Chinhengo, Shannon D. Anderson, Elsa Fiedrich, Deborah McNeil

**Affiliations:** ^1^ Faculty of Nursing, University of Calgary, Calgary, AB, Canada; ^2^ Department of Community Health Sciences, Cumming School of Medicine, University of Calgary, Calgary, AB, Canada; ^3^ Partnerships and Innovation, Acute Care Alberta, Calgary, AB, Canada; ^4^ Werklund School of Education, University of Calgary, Calgary, AB, Canada; ^5^ Alberta Health Services, Calgary, AB, Canada; ^6^ Parent Partner, Alberta Health Services, Calgary, AB, Canada; ^7^ Birth and Postpartum Doula, Edmonton, AB, Canada; ^8^ Covenant Health, Edmonton, AB, Canada; ^9^ Section of Newborn Critical Care, Department of Pediatrics, Cumming School of Medicine, University of Calgary, Calgary, AB, Canada

**Keywords:** knowledge translation, co-developed research, patient- and family-centered care, patient engagement, patient and public involvement, patient-oriented research

## Abstract

**Background:**

Co-developing research in partnership with patients and families is integral to Learning Health Systems (LHSs). These partnerships advance LHS objectives by (1) ensuring innovation is relevant to local contexts, (2) accelerating evidence into practice, and (3) improving services and outcomes that are meaningful to patients and families. Despite the importance of patient and family engagement in LHSs, strategies that guide researchers to build and sustain teams of patients, clinicians, and other partners are under-reported.

**Objective:**

We report actionable insights for co-developing research learned through our experience within Alberta's LHS.

**Context:**

Parents from a provincial advisory group in Alberta identified the need to evaluate parents' experiences with family-centered care in Neonatal Intensive Care Units (NICUs). In response, a research team of parent partners, researchers, and clinicians is co-developing a validated experience measure for parents in NICUs.

**Methods:**

During co-development, the research team engaged in reflective practice through semi-structured discussion informed by Schön's Reflection Model. Notes from the discussion were thematically analyzed to identify insights and research co-development strategies.

**Results:**

Three key insights and associated strategies were generated: (1) operationalizing co-development through a shared governance structure, terms of reference, and dedicated reflection; (2) adaptive approaches to team member involvement, renegotiating workflows, and addressing dissent; and (3) team evolution by nurturing reciprocity and utilizing existing partnerships to recruit members.

**Conclusion:**

We demonstrate how a team of patient partners, researchers, and clinicians can effectively co-develop research to address health system issues, and we present strategies to support patient-oriented research teams within LHSs.

## 1 Introduction

Healthcare systems internationally are striving to become Learning Health Systems (LHSs) to achieve high-quality, patient-centered care at reasonable costs through systematic and rapid implementation of evidence-based care (1–3). Numerous LHS definitions, models, theories, and frameworks have been developed since the concept was originally introduced in 2007 (3, 4). Most LHS frameworks emphasize cycles of data-to-knowledge-to-practice wherein research evidence is implemented into practice in a timely manner and then continuously evaluated to enable ongoing improvement and adaptation (4, 5). The continuous cycles of learning and improvement within LHSs are generally driven by communities of interest who are motivated to improve a central goal or issue (6). As such, the underlying system culture must be conducive to continuous learning and improvement, and a strong infrastructure is needed to support and accelerate the iterative process of moving knowledge into practice (6, 7).

Disciplines, professions, and knowledge users often exist in silos within healthcare systems; LHSs provide an effective model to strategically align groups towards collaboration on the complex challenges at the LHS' center (8). Within the transdisciplinary teams that drive LHSs, “patients, families, and community members” (PFC) are critical contributors to meaningful, patient-led systems change and are a core element of LHSs (4–6, 9).

The inclusion of PFC within a LHS (a) increases the relevance of research questions and findings to PFC, (b) enhances data that informs clinical decision-making, (c) improves research dissemination and uptake in clinical settings, and (d) positively impacts the overall research culture to be more inclusive, relevant, and patient-centered (10–12). Patient-oriented research (POR) provides a framework to integrate PFC into LHSs to ensure that learning and improvement within the system is informed by relevant patient priorities (5). A core component of POR is patient engagement which includes inclusivity, support, mutual respect, and co-build (13).

Malterud and Elvbakken (14) systematically reviewed outcomes and experiences reported in 17 health research studies engaging patients as co-researchers in priority-setting, planning, design, data collection, analysis, and reporting of research. Seven of the 17 studies specifically explored the shared experiences of patients involved as co-researchers as the primary focus of the manuscript; however, the authors found that despite the focus on the process of collaboration, only two studies reported tangible interactions within the research teams that facilitated collaboration (14). Furthermore, the reviewers noted that the scientific rigor of the studies reviewed may have been compromised to accommodate patient partners. This highlights a need to examine patient engagement strategies that balance the potential trade-offs between fostering rigorous research and integrating patient partners into research in an ethically responsible way.

Despite the increasing emphasis on integrating engaged and empowered patients within LHSs, existing research primarily focuses on the development and description of LHS theories and frameworks, rather than practical application (4). To enhance the theoretical and empirical knowledge base surrounding how co-developed research works, Dunn, Bhati et al. (15) recently completed a meta-synthesis of 35 case studies involving research co-development with patient partners in clinical and health services research settings. While the integration of case study findings resulted in a deeper understanding of the barriers, facilitators, and outcomes of co-developed research, the researchers noted that there remains a “how-to” knowledge gap regarding the tangible processes and activities undertaken to co-develop research (15). Specifically, Dunn and colleagues (15) highlight that more research and detailed reflection by research teams is needed to better understand how team relationships are formed and maintained, how communication is facilitated, and how specific team processes foster effective team functioning and engagement throughout a project.

This knowledge gap also exists in LHSs (16), indicating the need for a deeper understanding of patient-partner roles, activities, and tangible strategies to best support partnership with and learning from PFC (17–19). Thus, the objective of this manuscript is to report actionable key insights and practical strategies that facilitate co-development of research amongst a team of parent-partners, clinicians, and researchers within a LHS. These insights and strategies were identified through reflection on our team's experience with a novel, co-developed research project that is in-progress within a Canadian LHS.

## 2 Context

The research project was developed in collaboration with a former Strategic Clinical Network in the healthcare system of Alberta, Canada (20). Several parents from an existing patient and family advisory council, personally experienced with the Neonatal Intensive Care Unit (NICU), identified the need for a province-wide instrument to evaluate family experiences in Alberta NICUs. Existing instruments were site specific, not validated to measure the concept of experience, lacked information on measurement properties, and/or were not developed with input from families (21–23).

This call to action by parent advisors led to the creation of a research team to co-develop a validated instrument to measure family experiences in the NICU called the Neonatal Intensive Care Experience Reporting (NICER) Instrument. The research team includes two health services researchers, one doctoral student, two NICU clinicians, and three parent-partners with varied levels of past research experience. Several members of the team are also administrative leaders within the healthcare system. Consultation sessions with other key NICU knowledge users were integrated into the development of the NICER Instrument to provide broader perspectives and help ensure the instrument resonates with other parents and NICU clinicians. Three existing parent advisory groups and a provincial NICU committee collaborated on the project.

Co-development of the NICER Instrument is following a rigorous four-phase process to ensure a reliable, relevant, and valid experience measure is created. The project is currently in the last phase: assessing measurement properties in Alberta NICUs. Throughout each development phase, from formulating the research topic through to collecting data from NICUs, parent-partners have been co-leading the research. All decisions and processes have and will continue to prioritize the co-build principle of patient engagement with parent-partners working closely with researchers and clinicians throughout to identify priorities, co-develop knowledge, and generate solutions (13).

## 3 Methods

To identify key insights and practical strategies that facilitated progress of this POR project, our research team reflected as a group on the experience of working together. Reflection is a process of developing new understanding, knowledge, or meaning based on the intentional examination of experiences, actions, and outcomes (24–27). Within a LHS, critical reflection among teams is foundational to the learning and improvement that occurs (6). Reflection prompts teams to act based on what they learn from their actions (6).

Reflection as a research method is particularly effective in co-developed research because of the prioritization of reciprocity, emphasis on co-constructing knowledge, and focus on hearing and privileging the voices of all research team members (28). Even though our team engaged in reflection throughout the NICER Instrument co-development process, we held a purposeful reflective practice session informed by the work of Schön (25) to share and learn from our personal and collective experiences. This session occurred at the project's midpoint over a one-hour Zoom video call and centered on the experience and process of co-development, with the broad question, “What have we learned to date through the experience of working together as a team to co-develop the NICER Instrument?”.

Author JMW thematically analyzed the detailed written notes from the session following Braun and Clark's (29) six phases of thematic analysis, including (1) familiarizing oneself with the data; (2) generating initial codes; (3) collapsing codes into broader themes; (4) reviewing the themes and recoding as needed; (5) naming the themes; and (6) writing the manuscript. Two follow-up virtual (Zoom) team meetings were held to concurrently member-check and triangulate the identified themes. The team collaborated on naming each theme to ensure collective thoughts were meaningfully captured. Finally, our team provided input into the interpretation of the key insights learned and created this manuscript together.

## 4 Findings

Engaging in reflective practice and subsequent analysis of our discussion enabled our team to identify three key insights and describe practical strategies supporting co-development of the NICER Instrument (Table 1). Each theme is described below.

**Table 1:** Key insight and practical strategies utilized by the NICER research team.

Key insight identified by NICER Research Team	Practical strategies used by NICER Research Team
Operationalizing Co-development	• Intentionally **creating a shared governance structure** for the project to ensure all relevant knowledge users are involved and had an opportunity to share their perspectives. • **Reflective team meetings** to facilitate feedback on the co-development process, adapt processes as needed, and enhance accountability. • **Ongoing development of a terms of reference** to outline the scope, responsibilities, decision-making processes, and involvement of research team members as the project evolves.
Adaptive Approaches	• Maintaining **flexible engagement options**, including meeting times, the use of phone and video meetings, addition of *ad hoc* meetings, and email updates. • **Renegotiating workflows** in response to contextual impacts and the priorities at each phase of the project. • **Dedicating time to work through dissent** through open dialogue within the research team to find a solution that meets the needs of all members.
Team Evolution	• **Utilizing existing networks to fill knowledge gaps** within the research team and broader shared governance structure to enhance project feasibility and sustainability of the NICER Instrument. • **Nurturing reciprocity** to recognize the value and importance of the knowledge that each research team member contributes to the project.

### 4.1 Operationalizing co-development

**Figure 1:**
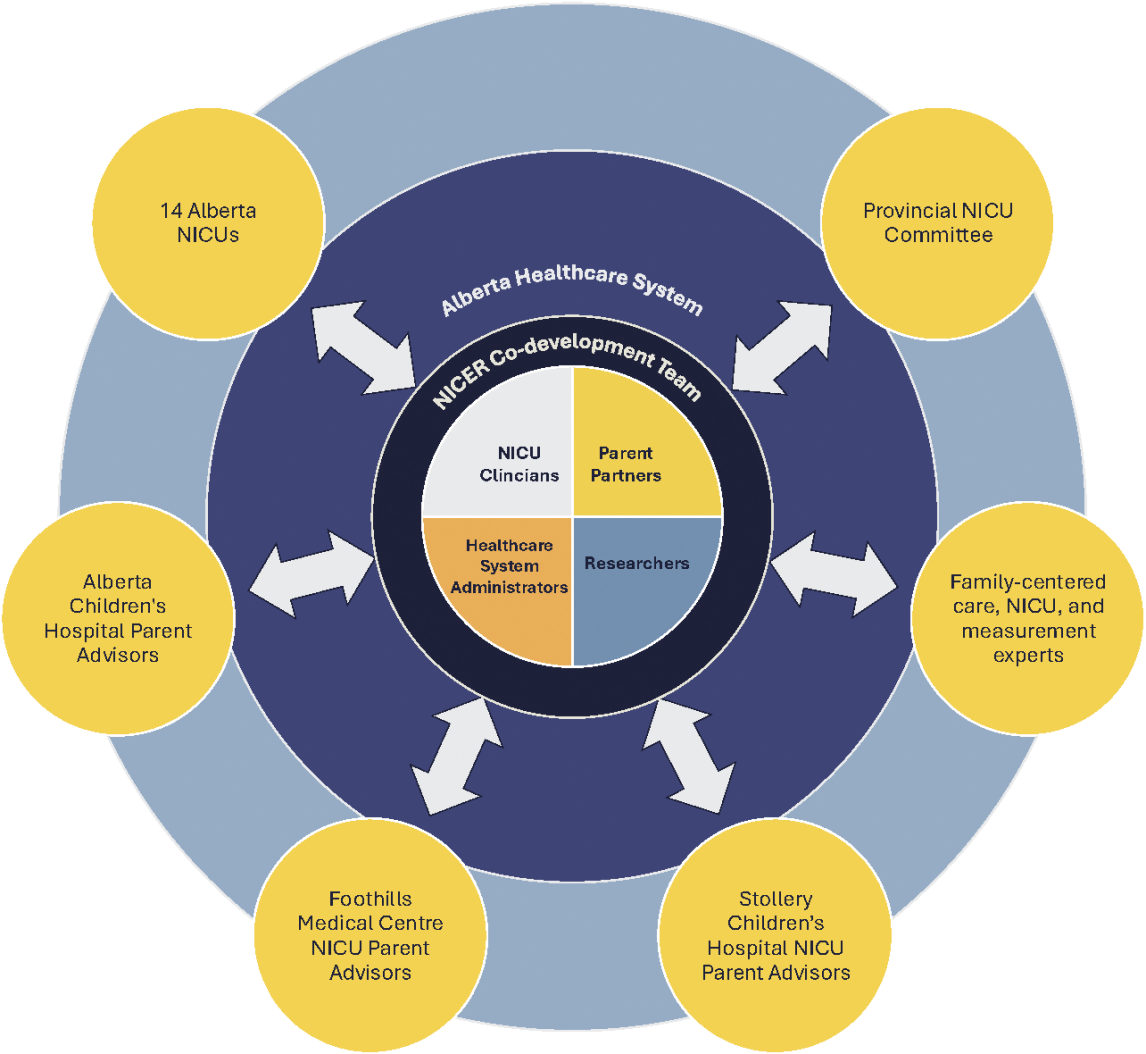
Shared governance structure utilized by the NICER research team.

One of the most salient aspects of the co-development process was the conscious dedication of time and dialogue to operationalize how the team would share leadership and decision-making. A *shared governance structure* was created to promote shared decision-making, collective accountability, and shared responsibility by configuring the relationships between individuals and groups involved in co-developing the NICER Instrument (Figure 1). At the center, key perspectives of research team members are equally weighted in four quadrants to reflect the shared power and decision-making inherent to our team's values and processes. All members share leadership and responsibility for decisions regarding design, data collection, analysis, and knowledge translation. Outer circles represent additional groups of key collaborators involved in the co-development process. Finally, the entire governance structure is situated within Alberta's healthcare system, demonstrating our team's embedded position and the potential impact of contextual factors.

Another output was a *modifiable terms of reference* document which provided a shared understanding of the project and related processes amongst all team members. One member noted, “Advisors may feel that things are over their head, and they don't understand potentially complex project components,” highlighting the importance of the document. Reflection and group discussions continue to determine if elements of the terms of reference need to be modified.

### 4.2 Adaptive approaches

Another team member stated, “Co-development is a culture shift and an opportunity for growth for research teams. This uncertainty and change from norms could be a challenge for those who are new to this type of work.” This comment led our team to reflect on the importance of remaining open to new ideas and adapting as needed.

Involvement in co-developed research can be time-consuming depending on engagement, expectations, and responsibilities outlined in the terms of reference. To facilitate involvement of all team members, the terms of reference enables us to remain *flexible* in our roles and responsibilities based on personal capacity.

Collaborating with advisory groups can introduce new ideas or processes, leading to *renegotiation of existing workflows*. One team member reflected on the importance of speaking with external advisors to consider how existing processes, particularly when conducting research in clinical environments, can be leveraged to minimize disruption caused by research. Despite our commitment to equality and collaborating to share power and responsibility within our team, some stages of the project required certain perspectives to be prioritized. For example, workflow was adapted to prioritize parent-partner voices while writing the NICER Instrument questions and clinician perspectives were prioritized when planning data collection processes.

While our team commended one another for the safe space to share and positive team culture, we reflected on the importance of remaining flexible in problem-solving and *addressing dissent* within the team. As one member stated, “When we hit a stumbling block or challenge, we kept going and listened to dissent. We didn't give in to the group majority, we respected diverse opinions and kept working at it until we found common ground that met the perspectives of all members of the project.” Our team worked through dissent during dedicated meetings to address issues. Providing dedicated time and space enabled each team member to share their perspective and led to increased mutual understanding. Relevant literature was found to guide decisions as needed and multiple iterations of challenging project components were reviewed until mutually satisfactory solutions were found. For example, during formulation of the NICER Instrument demographic questions, several meetings were needed to explore the differences between parent-partners' and researchers' priorities regarding the types of demographic information to collect that led to co-developing a set of questions that satisfied the priorities of both.

### 4.3 Team evolution

Following initial team meetings, additional members were *sought from existing networks*. Our team carefully reflected on whether the perspectives of any key knowledge users were missing, such as NICU clinicians, and then recruited them. Our team remained cognisant of whether it was being dominated by certain knowledge users who could assume power to drive decisions. Inclusion of multiple parent-partners who shared their perspectives and experiences helped mitigate this risk and is a strength of this project.

From the beginning, time was dedicated to building relationships within the team, creating a supportive team culture, and fostering commitment to the project. Throughout our time working together, our team supported each other through the co-developed research, but also through job transitions, life milestones, and even the arrival of a baby. As one team member stated, “The development of our team's culture has been important in building trust to ensure that it is a safe space, and the information shared will be heard.”.

Our team is committed to *nurturing reciprocity*. We actively recognize the important knowledge that each team member contributes and have carefully created a culture of safety and trust for team members to share their perspectives in a constructive, positive environment.

## 5 Discussion

The evidence generated by a LHS is inherently tied to the knowledge users within the system who contribute to the learning (16) and to the integration of PFC to enhance outcomes (4). However, there is a lack of detail and poor differentiation between the structures and the processes for partnering with PFC (16). Only a small proportion of LHSs involve learning with PFC through the formation of partnership, power-sharing, and co-producing knowledge. More commonly, LHSs learn from communities through data extraction (16). There is an expectation that involving PFC in LHSs must move PFC beyond being donors of data and towards being partners in the research process, however, there remains a lack of understanding regarding how to build and support these crucial partnerships (19).

Our experience co-developing research as a team was informed by theoretical principles of patient engagement (13), but the engagement and team building process evolved organically and without a pre-established set of strategies or structured guide. Our experience of getting to know one another, planning, and working together through decisions, cultivated our growth into a cohesive team. In contexts where there is no set blueprint for action and individuals must improvise, make decisions, and remain adaptive, engaging in reflection is an important practice to consolidate knowledge (25, 30).

Our identification of operationalizing co-development, adaptive approaches, and team evolution along with practical strategies to operationalize these concepts aligns with the need to change existing research culture and move patient engagement from rhetoric to a reality within LHSs (16). Much of the focus regarding the development of LHSs is centered on technological infrastructure to support iterative cycles of data collection and quality improvement (31). However, there is increasing recognition that social development within the system is also required (31). The culture of a group within a LHS promotes cohesion, norms, and social dynamics that motivate group members towards a shared objective (31). Practices such as developing group value statements and processes to work through tension contribute to a positive cultural infrastructure in LHSs (31). We fostered a culture of collaboration by developing a shared governance structure, terms of reference, and processes for shared decision-making. We also built shared values by taking time to bond as a team and get to know one another. These social encounters enabled us to nurture a sense of reciprocity and deeper collaboration through shared experiences related to both the research and our lives outside of the project (28).

As LHSs increasingly depend on interdisciplinary teams, understanding barriers and facilitators, ideal conditions, and proven strategies that support collaboration is valuable. Empirical evidence outlining factors and practices that influence the effectiveness of teams conducting scientific research exists (32, 33). Initially conceptualized in 2006, *team science* integrates individuals with diverse expertise and disciplinary backgrounds to address complex and multidimensional societal problems in scientific research environments (34). Features integral to successful science teams include (1) developing a shared vision, expectations, and role assignments within the team; (2) strategically recruiting team members to address team needs; (3) engaging in disagreements, continuing the dialogue, and working through issues; (4) strengthening team dynamics through effective communication; and (5) building personal connections within the team to share in the joy of science together (35).

The study of the effectiveness of science teams has been examined in the context of patient and community engagement to study the overlap between the science of team science and principles of patient engagement (34). Researchers may be applying team science strategies to research projects engaging PFC without realizing it (34), particularly in LHS where PFC are critical contributors. This is evidenced by our team's intuitive application of the above-mentioned team science features while unaware of their recommended employment.

### 5.1 Limitations

The findings shared in this manuscript should be interpreted in light of their limitations. The team reflective practice was not recorded (video or audio), possibly limiting the granularity of the data analyzed. However, the research team was part of the analysis, interpretation, and writing of this manuscript to ensure that the results and subsequent discussion aligned with their voiced experience and perspectives. Furthermore, while the research team engaged in transparent, stimulating, and reciprocal dialogue, it is possible that team members may not have shared all thoughts regarding the co-development experience.

The research team includes members from rural and urban areas, cis-gendered women, racialized people, and caregivers of children with disabilities, but we recognize our small team may not represent all perspectives within our research context. Finally, key insights and practical strategies reported in this manuscript are specific to one project and clinical context; the transferability of findings to other contexts requires further study. Even so, we think that sharing lessons from our experience may support others in their endeavors towards co-developed research, contributing to the evidence surrounding practical strategies and tangible guidance to support patient engagement in LHS research.

## 6 Conclusion

When undertaking research co-development, it is imperative to engage the perspectives of PFC who are impacted by healthcare practices, yet tangible strategies to achieve this goal are under-reported in the LHS literature. Using reflection, we identified actionable key insights of operationalizing co-development, using adaptive approaches, and fostering team evolution to support a team research approach. Real-world examples of practical strategies that facilitated our team's effective function are outlined in this manuscript and demonstrate the alignment between team science and POR research. Team science is a useful addition to guide and support POR in a LHS as it can address the existing how-to knowledge gap, potentially increasing the success of transdisciplinary research teams in LHSs.

## Data Availability

The datasets presented in this article are not readily available because original contributions are included in the manuscript but are available upon reasonable request. Further inquiries can be directed to the corresponding author. Requests to access the datasets should be directed to jacqueline.wilson@ucalgary.ca.
